# Second primary malignancies in patients with clinical T1bN0 esophageal squamous cell carcinoma after definitive therapies: supplementary analysis of the JCOG trial: JCOG0502

**DOI:** 10.1007/s00535-022-01870-y

**Published:** 2022-05-11

**Authors:** Seiichiro Mitani, Ken Kato, Hiroyuki Daiko, Yoshinori Ito, Isao Nozaki, Takashi Kojima, Masahiko Yano, Satoru Nakagawa, Masaki Ueno, Masaya Watanabe, Shigeru Tsunoda, Tetsuya Abe, Shigenori Kadowaki, Tomohiro Kadota, Keita Sasaki, Ryunosuke Machida, Yuko Kitagawa

**Affiliations:** 1 Department of Clinical Oncology, Aichi Cancer Center Hospital, 1-1 Kanokoden, Chikusa-ku, Nagoya, Aichi 464-8681 Japan; 2 Department of Medical Oncology, Faculty of Medicine Kindai University, 377-2 Onohigashi, Osaka-sayama, Osaka, 589-8511 Japan; 3 Department of Head and Neck, Esophageal Medical Oncology, National Cancer Center Hospital, Tokyo, Japan; 4 Esophageal Surgery Division, National Cancer Center Hospital, Tokyo, Japan; 5 Department of Radiation Oncology, Showa University School of Medicine, Tokyo, Japan; 6 Department of Gastroenterological Surgery, National Hospital Organization Shikoku Cancer Center, Matsuyama, Japan; 7 Department of Gastroenterology and Gastrointestinal Oncology, National Cancer Center Hospital East, Kashiwa, Japan; 8 Department of Surgery, Osaka International Cancer Institute, Osaka, Japan; 9 Department of Surgery, Niigata Cancer Center Hospital, Niigata, Japan; 10 Department of Gastroenterological Surgery, Toranomon Hospital, Tokyo, Japan; 11 Department of Gastroenterological Surgery, Shizuoka General Hospital, Shizuoka, Japan; 12 Department of Surgery, Graduate School of Medicine, Kyoto University, Kyoto, Japan; 13 Department of Gastroenterological Surgery, Aichi Cancer Center Hospital, Aichi, Japan; 14 Department of Gastroenterology and Endoscopy, National Cancer Center Hospital East, Kashiwa, Japan; 15 Japan Clinical Oncology Group Operations Office, National Cancer Center Hospital, Tokyo, Japan; 16 Japan Clinical Oncology Group Data Center, National Cancer Center Hospital, Tokyo, Japan; 17 Department of Surgery, Keio University School of Medicine, Tokyo, Japan

**Keywords:** Esophageal cancer, Second malignancies, Esophagectomy, Definitive chemoradiotherapy

## Abstract

**Background:**

Previous studies have suggested that patients with esophageal squamous cell carcinoma (ESCC) are still at a high risk of developing second primary malignancies (SPMs) after definitive therapies. We evaluated the development of SPMs and explored its risk factors in patients with clinical T1bN0 ESCC.

**Methods:**

JCOG0502 prospectively compared esophagectomy with definitive chemo-radiotherapy for clinical T1bN0 ESCC. Here, we reviewed all JCOG0502 patients’ data for SPMs and investigated the risk factors for SPMs using uni-variable and multivariable analyses by Fine and Gray model.

**Results:**

Among 379 enrolled patients, 213 underwent esophagectomy and 166 received chemo-radiotherapy. Patient characteristics were male (85%); median age [63 (range 41–75) years; location of the primary tumor (upper/middle/lower thoracic esophagus, 11%/63%/27%, respectively]; alcohol consumption history (79%); smoking history (66%); prevalence of no/several/many/unknown Lugol-voiding lesions (LVLs) (45%/36%/8%/11%, respectively). In a median follow-up of 7.1 years, 118 SPMs occurred in 99 (26%) patients. Cumulative incidences of SPMs after 3, 5, and 10 years were 9%, 15%, and 36%, respectively. The most common primary tumor sites were the head and neck (35%), stomach (20%) and lungs (14%). In multivariable analyses, compared to no LVLs, several LVLs [hazard ratio (HR) 2.24, 95% confidential interval (CI) 1.32–3.81] and many LVLs (HR 2.88, 95% CI 1.27–6.52) were significantly associated with the development of SPMs. Sixteen patients died due to the SPMs.

**Conclusion:**

The incidence of SPMs was high. The presence of LVLs, which was a predictive factor for SPMs, may be useful for surveillance planning.

**Supplementary Information:**

The online version contains supplementary material available at 10.1007/s00535-022-01870-y.

## Introduction

Esophageal cancer is one of the most fatal diseases worldwide, mainly because of its high-grade malignancy [1]. In Asia, squamous cell carcinoma is the predominant histological type of esophageal cancer [2, 3]. Multiple squamous cell carcinomas frequently arise in the upper aero-digestive tract. The carcinogenic effects of tobacco and alcohol on the other parts of the aero-digestive tract, lead to the frequent occurrence of simultaneous or metachronous cancer development, particularly in the head and neck region and esophagus. This phenomenon is referred to as “field cancerization” [4, 5]. Thus, as recommended in guidelines, screening for double cancer should be performed during pretreatment examination of esophageal cancer [6]. In addition, several studies have suggested a remaining risk of second primary malignancies (SPMs) even after the completion of treatment for esophageal cancer. Several retrospective studies enrolled patients with esophageal cancer who underwent esophagectomy or received definitive chemo-radiotherapy, and demonstrated a high mortality following the development of SPMs [7–10]. Pooled analyses of multiple cancer registries and a population-based study surveying a large cohort of patients with esophageal cancer reported a significantly increased risk of developing metachronous SPMs [11–13].

We previously conducted a retrospective analysis of 758 patients with esophageal cancer and found an increased incidence of SPMs in patients with esophageal cancer even after definitive treatment [14]. Furthermore, early clinical stage was identified as a significant factor for the incidence of SPMs. This can be explained by the fact that SPMs can occur during a longer survival period in patients with early-stage cancers because recent advances in multimodal treatment strategies have contributed to the increased survival rate. Therefore, there is need for special attention regarding the possibility of SPMs developing in patients with early-stage esophageal cancer. However, the above-mentioned previous studies might have underestimated the incidence of SPMs due to the retrospective nature of the study. In addition, the risk of SPMs after treatment for early-stage esophageal cancer has not been fully investigated because most esophageal cancers are detected in the advanced stage. Smoking and alcohol consumption are well-known risk factors for the development of esophageal squamous cell carcinoma (ESCC) and head and neck cancers [15–17]. High-grade dysplasia and squamous cell carcinoma lack glycogen, are visible as void of Lugol staining in chromo-endoscopy, following iodine dye staining. The visible pattern of Lugol-voiding lesions (LVLs) can serve as an indicator of the risk of both esophageal cancer and head and neck cancers [18–20]. LVLs were also reported to be useful to predict the development of SPMs [19, 21]. We hypothesized that these risk factors are also associated with the incidence of SPMs.

JCOG0502, a multicenter phase III trial, compared esophagectomy with definitive chemo-radiotherapy in clinical T1bN0 ESCC [22]. This trial enrolled and prospectively examined 379 patients, to show the non-inferiority of chemo-radiotherapy compared with surgical resection. The objective of the present study was to evaluate the development of SPMs and explore its risk factors in patients with clinical T1bN0 ESCC using prospective data from JCOG0502.

## Methods

### Study design and patients

The details of JCOG0502 have been described elsewhere [22]. The main eligibility criteria were as follows: (1) histologically proven thoracic esophageal squamous cell, adeno-squamous or basaloid cell carcinoma; (2) clinical stage T1bN0M0 based on the 7th UICC-TNM classification; (3) age from 20 to 75 years; (4) Eastern Cooperative Oncology Group (ECOG) performance status (PS) of 0 or 1; (5) no prior therapy for esophageal cancer; (6) adequate organ function; and (7) without sever comorbidities. Screening tests for other active malignancies were conducted using upper endoscopy, computed tomography (CT) scans. Positron emission tomography or otolaryngological examination was not mandatory and performed according to investigator’s choice. All patients were informed of the randomized nature of the study. Only when patients refused randomization and consented to non-randomized parts of the trial, they were assigned to surgery or chemo-radiotherapy, as the patients and their oncology team decided. Esophagectomy with 2- or 3-field lymph node dissection was performed in the surgical arm. In the chemo-radiotherapy arm, cisplatin 70 mg/m^2^ (days 1 and 29) and 5-fluorouracil 700 mg/m^2^/day (days 1–4, 29–32) combined with 60 Gy/ 30 fr radiotherapy were delivered. Written informed consent was obtained from all the patients prior to enrollment. The study protocol of the trial was approved by the institutional review boards of all institutions. The study was conducted in accordance with the principles of the Helsinki Declaration of 1964 and its later amendments and registered in the clinical trial database (UMIN000000551). Using data from all patients enrolled in JCOG0502, we performed additional analyses for SPMs.

### Follow-up protocol

In the surgical arm, CT scans and tumor marker testing, such as carcinoembryonic antigen and squamous cell carcinoma antigen testing, were performed every 3 months in the first year, every 4 months in the second year, and every 6 months in the third, fourth, and fifth years after completion of surgical resection. Esophagogastroduodenoscopy was performed at the discretion of the investigators. In the chemo-radiotherapy arm, endoscopic examination was mandatory in addition to tumor marker testing, in similar time intervals as CT scan.

### Definitions and statistical considerations

SPMs were defined as malignancies developing in organs other than the esophagus after enrollment in JCOG0502. SPMs in the esophagus were excluded from this study. Patient characteristics, such as age, body mass index (BMI), history of smoking, and alcohol use, were based on data at the time of enrollment in JCOG0502. LVLs were assessed in the noncancerous esophageal mucosa using electronic images of endoscopic examinations before the initiation of treatment. Three experienced endoscopists who were blinded to the clinical data reviewed the images centrally. Lugol-voiding pattern was graded according to the maximum number of small LVLs in at least one endoscopic field of view as follows: grades A, B, and C referred to no; several (1 to 9); and many (≥ 10) small LVLs, respectively [19].

The cumulative incidence function for SPMs was estimated using death as a competing risk. Since mortality affects the occurrence of SPMs and can be a competing risk, uni-variable and multivariable analyses using the Fine and Gray model were performed to investigate the risk factors for SPMs [23]. The hazard ratio (HR) and its 95% confidence interval (CI) were calculated. From a clinical standpoint, the following 10 variables were selected: age (≥ 65 vs. < 65 years), sex (male vs. female), ECOG-PS (0 vs. 1), BMI (≥ 25 vs. < 25 sq/m^2^), history of alcohol consumption (no vs. 0–25 mL of ethanol per day vs. vs. ≥ 25 mL of ethanol per day), history of smoking (no vs. 1–20 cigarette per day vs. vs. ≥ 20 cigarette per day), LVLs (grade A vs. B vs. C), location of the primary tumor (upper thoracic esophagus vs. middle thoracic esophagus or lower thoracic esophagus), primary tumor length (≥ 4 vs. < 4 cm), and study treatment arm (surgery vs. chemo-radiotherapy). The cut-off value of BMI was determined according to World Health Organization (WHO) criteria, and the cut-off values of alcohol consumption and smoking were set based on previous reports [24, 25]. All tests were two-sided, and a *p* value < 0.05 was considered statistically significant. In the case of using a variable selection procedure, backward elimination method with a *p* value < 0.10 was used in multivariable analyses. Statistical Analysis Software (SAS) version 9.4 (SAS Institute, Cary, NC) was used for all statistical analyses.

## Results

### Patient characteristics

A total of 379 patients were registered in the JCOG0502 between December 2006 and February 2013. The median observation time was 7.1 years (range 0.0–11.0 years). The patient backgrounds are summarized in Table 1. The median age was 63 years (range, 41–75 years), and 323 (85%) patients were male. Among the randomized patients, four were assigned to surgery (Cohort A) and seven to chemo-radiotherapy (Cohort B). Regarding the patient-preference arm, 209 and 159 underwent surgery (Cohort C) and chemo-radiotherapy (Cohort D), respectively. Information on history of alcohol or smoking, or prevalence of LVLs was not obtained from all patients since these were not mandatory investigational items at the time of enrollment.

**Table 1:** Patient characteristics

	Patients (*n* = 379)
Age, years	
Median (range)	63 (41–75)
Sex	
Male	323 (85%)
Female	56 (15%)
Primary tumor location in the esophagus	
Upper thoracic esophagus	41 (11%)
Middle thoracic esophagus	237 (63%)
Lower thoracic esophagus	101 (27%)
Body mass index, sq/m^2^	
< 25	306 (81%)
≥ 25	73 (19%)
History of smoking	
Yes	251 (66%)
No	119 (31%)
Unknown	9 (2%)
History of alcohol consumption	
Yes	300 (79%)
No	48 (13%)
Unknown	31 (8%)
LVLs	
A	170 (45%)
B	136 (36%)
C	30 (8%)
Unknown	43 (11%)
Length of primary tumor	
< 4 cm	253 (67%)
≥ 4 cm	126 (33%)
Treatment modality	
Esophagectomy	213 (56%)
Chemo-radiotherapy	166 (44%)

*LVLs* Lugol-voiding lesions

### Incidences of SPMs

**Fig. 1:**
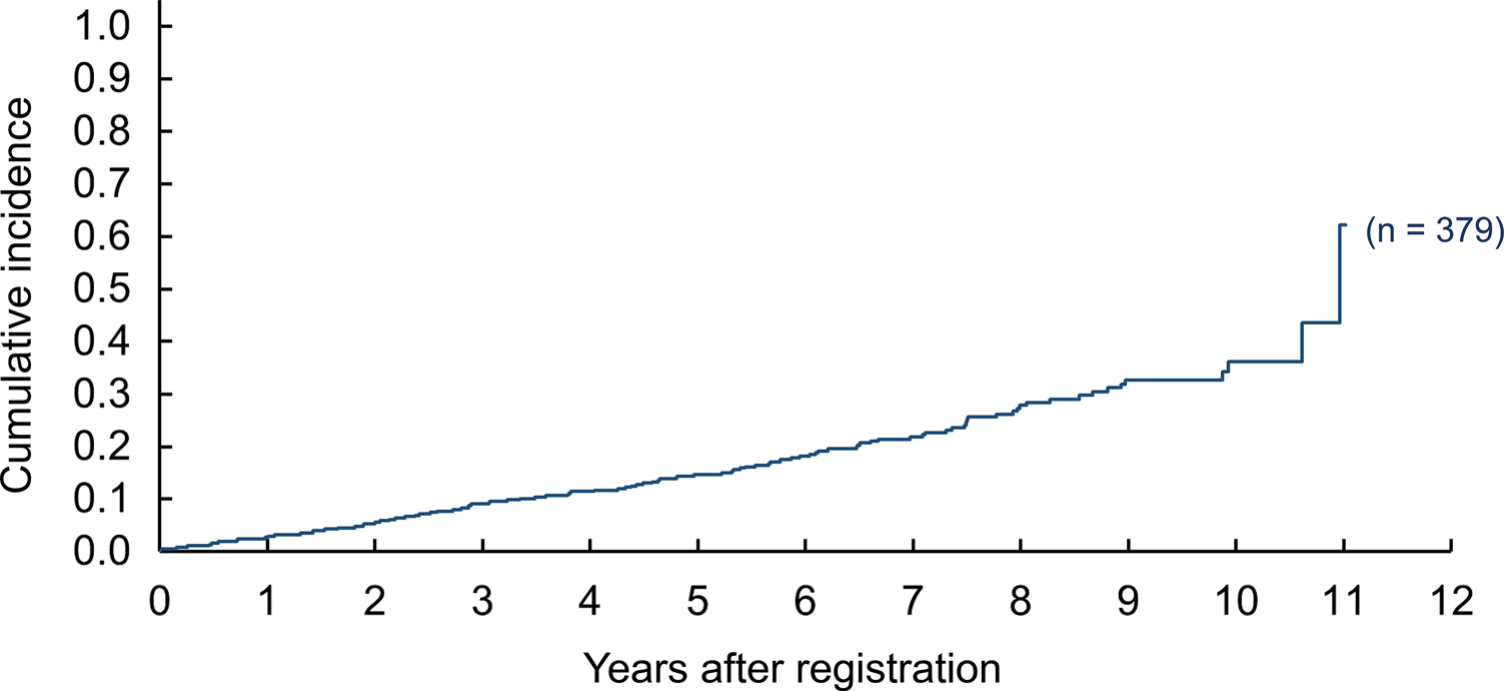
Cumulative incidence of second malignancies

A total of 118 SPMs were observed in 99 (26%) patients. Cumulative incidences after 3, 5, and 10 years were 9.0% (95% CI: 6.4–12.2); 14.7% (95% CI: 11.3–18.5); and 36.1% (95% CI: 28.9–43.5), respectively (Fig. 1). The most common primary tumor sites were the head and neck including five thyroid cancers (*n* = 41), stomach (*n* = 24), lung (*n* = 17), urinary tract (*n* = 10), colon and rectum (*n* = 9), pancreas (*n* = 3), liver (*n* = 3), and leukemia (*n* = 3) (Table 2). Cumulative incidences of head and neck cancers after 3, 5, and 10 years were 3.7% (95% CI 2.1–6.0), 5.3% (95% CI 3.4–7.9), and 11.5% (95% CI 7.9–15.8), respectively.

**Table 2:** Site of second primary malignancies

Site of second primary malignancies	*n* (%^a^)
Head and neck	41 (35%)
Stomach	24 (20%)
Lung	17 (14%)
Urinary tract	10 (9%)
Colorectal	9 (8%)
Pancreas	3 (3%)
Liver	3 (3%)
Leukemia	3 (3%)
Others	8 (7%)

^a^Of the denominator was 118 sites (in 99 patients with second primary malignancies)

Among head and neck cancers (excluding thyroid cancer), 29 (81%), 4 (11%), and 2 (6%) were detected at endoscopic examination, CT scan, and regular otolaryngological examination, respectively. The development of tumor-related symptoms triggered the detection of only one case of head and neck cancer. Consequently, 26 (72%), 8 (22%), and 2 (6%) cancers were diagnosed with clinical stages 0–I, II–III, and IV, respectively. All stomach cancers were detected at endoscopic examination and with clinical stages 0–I. Among lung cancers, 14 (93%) were detected by CT scans. The remaining one (7%) developed symptoms and was diagnosed with lung cancer. Ten (67%), four (27%), and one (7%) cancer cases were diagnosed with clinical stages 0–I, II–III, and IV, respectively.

### Death due to SPMs

Sixteen patients died due to SPMs. Among the five most common types of SPMs, there were four and two deaths due to head and neck and lung cancers, respectively. However, there was no death following stomach cancer. Three, two, and one death occurred among patients with pancreatic cancer; leukemia; and myelodysplastic syndrome, colorectal cancer, tracheal cancer, breast cancer, and renal pelvis cancer, respectively.

### Clinicopathological factors predicting the development of SPMs

The results of SPM risk analysis are presented in Table 3. Among 284 patients who had no missing data regarding the baseline background, both uni-variable and multivariable analyses revealed that the presence of LVLs was significantly associated with the development of SPMs. In the multivariable analysis, compared with no LVLs the HRs of several and many LVLs were 2.24 (95% CI 1.32–3.81) and 2.88 (95% CI 1.27–6.52), respectively. Multivariable analyses including all 379 patients were also conducted by analyzing missing data as a factor of missing value, which showed that compared to no LVLs, several (HR: 2.01, 95% CI 1.25–3.22) and many LVLs (HR: 2.44, 95% CI 1.13–5.25) were significant factors (Supplementary Table 1). Furthermore, a variable selection procedure also reproduced the results which revealed that the pattern of LVLs was significant (Supplementary Table 2). According to the grade of LVLs (A vs. B vs. C), the cumulative incidences of SPMs after 3, 5, and 10 years were 4.1% (95% CI 1.8–7.9) vs. 11.9% (95% CI 7.1–18.0) vs. 13.3% (95% CI 4.1–28.1); 7.2% (95% CI 3.9–11.7) vs. 20.0% (95% CI 13.7–27.2) vs. 26.7% (95% CI 12.3–43.4); and 31.2% (95% CI 19.7–43.4) vs. 41.2% (95% CI 31.0–51.1) vs. 33.9% (95% CI 17.4–51.2), respectively (Supplementary Fig. 1).

**Table 3:** Univariable and multivariable analyses of second primary malignancies (*n* = 284 patients)

Factors	Univariable analysis			Multivariable analysis		
	HR	95% CI	*p*	HR	95% CI	*p*
Age, years						
vs. < 65						
≥ 65	1.05	0.67–1.63	0.84	0.98	0.59–1.61	0.92
Gender						
vs. Female						
Male	1.28	0.66–2.48	0.47	1.23	0.56–2.71	0.61
ECOG-PS						
vs. 0						
1	1.07	0.12–9.83	0.95	1.00	0.10–10.48	1.00
Body mass index, sq/m^2^						
vs. < 25						
≥ 25	0.75	0.39–1.44	0.38	0.77	0.40–1.50	0.44
Smoking						
vs. 0 cigarette per day						
1–20	1.15	0.68–1.95	0.61	1.30	0.72–2.34	0.38
≥ 20	1.46	0.81–2.64	0.21	1.92	0.99–3.75	0.06
Alcohol consumption						
vs. 0 mL of ethanol per day						
0–25	0.75	0.36–1.56	0.44	0.75	0.32–1.77	0.51
≥ 25	0.85	0.47–1.55	0.60	0.63	0.31–1.30	0.21
Location of primary site						
vs. Ut						
Mt	1.62	0.64–4.07	0.31	1.91	0.72–5.08	0.20
Lt	1.41	0.53–3.79	0.49	1.33	0.49–3.62	0.57
LVLs						
vs. A						
B	2.10	1.30–3.39	0.003	2.24	1.32–3.81	0.003
C	2.57	1.15–5.72	0.02	2.88	1.27–6.52	0.01
Length of primary lesion						
vs. < 4 cm						
≥ 4 cm	1.38	0.88–2.17	0.16	1.25	0.77–2.03	0.37
Treatment modality						
vs. Surgery						
CRT	1.44	0.92–2.24	0.11	1.68	0.97–2.91	0.06

The patients included in these analyses had no missing data regarding the baseline background

*HR* hazard ratio, *CI* confidence interval, *ECOG-PS* Eastern Cooperative Oncology Group performance status, *Ut* upper thoracic esophagus, *Mt* middle thoracic esophagus, *Lt* lower thoracic esophagus, *LVLs* Lugol-voiding lesions, *CRT* chemo-radiotherapy

The association between SPMs and LVLs was observed in head and neck cancer (Supplementary Table 3). In the multivariable analysis including 284 patients whose baseline background data were not missing, compared to no LVLs, the HRs of several and many LVLs were 8.92 (95% CI 2.49–32.02) and 18.97 (95% CI 3.86–93.17), respectively. Meanwhile, the risk of other malignancies, such as gastric and lung cancers, did not increase in patients with LVLs (Supplementary Table 4). Interestingly, gastric cancer occurred more frequently in the chemo-radio-therapy arm (4% vs. 10%, HR 2.78, 95% CI 1.20–6.42).

## Discussion

This study investigated the development of SPMs in patients with clinical T1bN0 ESCC who were enrolled in JCOG0502. To the best of our knowledge, this is the first study to evaluate a second cancer incidence in esophageal cancer patients who underwent surgery or chemo-radiotherapy using data from a study where the patients were prospectively followed up according to a protocol-specified schedule. Although the Japanese Esophageal Cohort (JEC) study also evaluated SPMs in a prospective setting, patients with esophageal cancer who were treated with endoscopic resection were included [19, 21]. Besides, our study had a larger sample size and longer follow-up period.

In our study, a high incidence of SPMs of 26% was observed, reinforcing the importance of SPMs detection in improving survival outcomes in patients with early-stage ESCC. Among the retrospective studies that assessed SPMs after treatment for esophageal cancer, Sato et al. reported SPM as the most common cause of death in patients with thoracic esophageal cancer whose initial surgically-resected lymph nodes tested negative [7]. In addition, the JCOG9708, a single-arm phase II clinical trial enrolled the same population as JCOG0502 and evaluated chemo-radiotherapy in patients with stage I ESCC [26]. In that study, the proportion of SPMs was reported to be 25%, which is quite similar to that of our study. The prevalence of SPMs was also comparable to those of previous reports, showing that the common primary tumor sites of SPMs are the aero-digestive tract organs, such as the head and neck, lung, and stomach [7–9, 14, 19]. In particular, the incidence of head and neck cancer was high, which supports the well-established “field cancerization” concept.

Seventeen of 99 (17%) patients who developed SPMs died. Given that the incidence of SPMs was high during the follow-up period, the survival of a considerable number of patients irrespective of SPM in the study population could be explained by the follow-up strategies. In JCOG0502, the protocol specified endoscopic examinations or CT scans during follow-up periods to enable early diagnosis of SPMs, with resultant relatively good prognosis. Of the three most common types of SPMs, 72%, 100%, and 93% were head and neck, stomach, and lung cancers, respectively, detected at clinical stages of 0–I. The rate of early detection in our study was comparable to or higher than that of the report by Yamaguchi, et al. showing 75%, 92%, and 60% were head and neck, stomach, and lung cancers, respectively, detected at clinical stages of 0–I [9]. A Japanese multicenter study including 77 specialized hospitals previously investigated the timing of follow-up for esophageal cancer patients after curative surgery or definitive chemo-radiotherapy in clinical practice [27]. The outcome of this study demonstrated that most hospitals monitored their patients for at least 5 years in a routine follow-up, with an exceptionally high frequency of follow-up in the first 3 years after treatments, using upper gastrointestinal endoscopy or CT scan. Nonetheless, this study also showed a tendency toward a decreased follow-up frequency for the assessment of patients with clinical stages 0/I disease compared with patients with stages II–IV disease. Approximately 30–40% of patients with clinical stages 0/I underwent an annual or fewer numbers of upper gastrointestinal endoscopy and CT scan. This could be reasonable because patients with early-stage cancer are less likely to experience cancer recurrence. However, it is plausible to consider that particular attention need be paid not only to esophageal cancer recurrence but also to the high incidence of SPMs; thus, more intensive, and frequent follow-ups might also be critical in patients with stage I esophageal cancer. In the JEC study, an otolaryngologist examined head and neck regions at 12 months of intervals in addition to upper gastrointestinal endoscopy at 6 months of intervals. As a result, 100% of second primary head and neck cancers, which were higher than that of our study, were detected as superficial cancer, and there was no death due to head and neck cancers [28].

Meanwhile, cancer screening requires efficient and cost-effective approaches. In this regard, the identification of risk factors is useful, and that motivated us to conduct this study. Multivariable analyses revealed an association between the development of SPMs and Lugol-voiding patterns. The high prevalence of LVLs has been linked to inactive aldehyde dehydrogenase type 2 (ALDH2) phenotypes [29]. ALDH2 plays a significant role in alcohol metabolism, especially in the degradation of carcinogenic acetaldehyde. Therefore, inactive ALDH2 phenotypes lead to the accumulation of acetaldehyde; and the direct exposure of acetaldehyde to esophageal or oral epithelium is one of the mechanisms accounting for carcinogenesis in the esophagus and head and neck regions [30]. *TP53* mutations are frequently observed in the esophageal epithelium with high severity of LVLs grade [21]. Moreover, a recent study indicated that the replacement of normal esophageal mucosa with pathogenic mutant clones increased with age and was promoted by smoking and drinking [31]. These data are considered to support the concept of “field cancerization.” They could also explain the extremely high HR for the developments of head and neck cancers in patients with LVLs and contribute to the association between LVLs and the development of SPMs in this study. Meanwhile, the risk of SPMs other than head and neck cancers did not increase in patients with LVLs. This might suggest the requirement of intensive follow-up for head and neck regions especially in patients with LVLs. Additionally, since abstinence from drinking reportedly reduced cancer development even in patients with multiple LVLs, the identification of risk factors is vital in guiding patient education, particularly for those at a high risk [21].

In the analyses of prognostic factors, a higher incidence of SPMs in the chemo-radiotherapy arm was observed. However, in JCOG0502, most patients received the study treatment in a non-randomized patient-preference manner. Therefore, there was an imbalance in patient characteristics between the surgical and chemo-radiotherapy arms, which made it difficult to evaluate whether treatment modalities affected the occurrence of SPMs. Furthermore, esophagogastroduodenoscopy was not mandatory in the surgical arm, which could have contributed to differences in detecting gastric cancer. Compared with the surgical arm, more than double stomach cancer incidence rates were observed in the chemo-radiotherapy arm. Although data on the interval of esophagogastroduodenoscopy were not obtained in the present study, it was reported that approximately 10% or less of the hospitals continued follow-up after esophagectomy without using upper gastrointestinal endoscopy [27]. Our results may indicate the importance of regular upper gastrointestinal endoscopy to detect stomach cancer even when esophagectomy is performed. Previous studies have suggested an elevated risk of SPM in patients who received radiotherapy or chemotherapy [11, 12]. However, further research is warranted in this regard.

There are some limitations to the present study. First, the data on social history or LVLs were missing in some patients because the data were not mandatory at enrollment. Iodine staining is sometimes irritable and time-consuming. Since recent studies have reported that image-enhanced endoscopy can replace Lugol’s iodine staining, the optimal method should be determined in further research [32]. Moreover, alcohol consumption or smoking habit during the study treatments and follow-up periods was not analyzed. In addition, the median observation period of 7.1 years might not be long enough for the evaluation of late carcinogenic effects induced by chemotherapy or radiotherapy. However, despite these limitations, the present study contributes to follow-up strategies after curative treatments for early-stage esophageal cancer. SPMs, such as head and neck cancers and stomach cancer, developed with high cancer incidence, suggesting that regular otolaryngological examination or endoscopic examination is needed especially in patients at high risk to improve early detection rates of SPMs and prevent death due to SPMs.

In summary, our data show that the incidence of SPMs was high, indicating that careful attention to monitoring and management is crucial after treatment completion. However, the establishment of optimal surveillance planning is undoubtedly required. We plan to further study of SPMs using combined data with other studies, such as the JCOG0508 [33] and the JEC study [21], including patients with early-stage esophageal cancer.

## Supplementary Information

Below is the link to the electronic supplementary material.

Supplementary file1 (DOCX 53 KB)

Supplementary file2 (PPTX 69 KB)
