# Supplementary material for: Second primary malignancies in patients with clinical T1bN0 esophageal squamous cell carcinoma after definitive therapies: supplementary analysis of the JCOG trial: JCOG0502
Source: J Gastroenterol. 2022 May 11;57(7):455–63. doi: 10.1007/s00535-022-01870-y (PMC9232445; doi:10.1007/s00535-022-01870-y)
Supplement: Supplementary file 1 — Supplementary file1 (DOCX 53 KB) [file 535_2022_1870_MOESM1_ESM.docx]

**Supplementary Table 1. Univariate and multivariable analyses of second primary malignancies (n = 379 patients)**

| **Factors** |  | **Univariate analysis** | | | **Multivariate analysis** | | |
| --- | --- | --- | --- | --- | --- | --- | --- |
|  |  | **HR** | **95% CI** | ***p*** | **HR** | **95% CI** | ***p*** |
| **Age, years**  **vs. < 65** | **≥ 65** | **1.08** | **0.73–1.60** | **0.70** | **1.06** | **0.69–1.62** | **0.81** |
| **Gender**  **vs. female** | **Male** | **1.38** | **0.74－2.57** | **0.31** | **1.45** | **0.67－3.14** | **0.34** |
| **ECOG PS**  **vs. 0** | **1** | **1.11** | **0.12－10.15** | **0.93** | **0.76** | **0.07－7.93** | **0.82** |
| **Body mass index**  **vs. < 25, sq/m^2^** | **≥ 25** | **0.70** | **0.40－1.23** | **0.21** | **0.70** | **0.39－1.25** | **0.23** |
| **Smoking**  **vs. 0 cigarette per day** | **1－20**  **≥ 20**  **missing** | **1.17**  **1.73**  **1.26** | **0.71－1.91**  **1.02－2.93**  **0.57－2.80** | **0.54**  **0.04**  **0.57** | **1.22**  **1.95**  **1.75** | **0.71－2.12**  **1.06－3.56**  **0.70－4.35** | **0.47**  **0.03**  **0.23** |
| **Alcohol consumption**  **vs. 0 mL of ethanol per day** | **0－25**  **≥ 25**  **missing** | **0.71**  **0.85**  **0.45** | **0.35－1.43**  **0.48－1.49**  **0.19－1.07** | **0.33**  **0.56**  **0.07** | **0.63**  **0.57**  **0.32** | **0.28－1.44**  **0.28－1.14**  **0.10－1.00** | **0.28**  **0.11**  **0.05** |
| **Location of primary site**  **vs. Ut** | **Mt**  **Lt** | **1.44**  **1.30** | **0.69－3.00**  **0.59－2.29** | **0.34**  **0.52** | **1.59**  **1.21** | **0.73－3.48**  **0.53－2.76** | **0.25**  **0.64** |
| **LVLs**  **vs. A** | **B**  **C**  **missing** | **1.95**  **2.00**  **1.55** | **1.25－3.05**  **0.94－4.25**  **0.79－3.04** | **0.003**  **0.07**  **0.20** | **2.01**  **2.44**  **1.79** | **1.25－3.22**  **1.13－5.25**  **0.90－3.57** | **0.004**  **0.02**  **0.10** |
| **Length of primary lesion**  **vs. <4 cm** | **≥ 4cm** | **1.11** | **0.74－1.68** | **0.62** | **1.03** | **0.68－1.58** | **0.88** |
| **Treatment modality**  **vs. Surgery** | **CRT** | **1.73** | **1.17－2.56** | **0.006** | **1.85** | **1.19－2.88** | **0.007** |

*HR* hazard ratio, *CI* confidence interval, *ECOG PS* Eastern Cooperative Oncology Group performance status, *Ut* upper thoracic esophagus, *Mt* middle thoracic esophagus, *Lt* lower thoracic esophagus, *LVLs* Lugol-voiding lesions, *CRT* chemoradiotherapy

**Supplementary Table 2. Univariable and multivariable analyses of second primary malignancies using a variable selection procedure (n= 284 patients)^a^**

| **Factors** |  | **Univariate analysis** | | | **Multivariate analysis** | | |
| --- | --- | --- | --- | --- | --- | --- | --- |
|  |  | **HR** | **95% CI** | ***p*** | **HR** | **95% CI** | ***p*** |
| **Age, years**  **vs. < 65** | **≥ 65** | **1.05** | **0.67–1.63** | **0.84** |  |  |  |
| **Gender**  **vs. female** | **Male** | **1.28** | **0.66－2.48** | **0.47** |  |  |  |
| **ECOG PS**  **vs. 0** | **1** | **1.07** | **0.12－9.83** | **0.95** |  |  |  |
| **Body mass index, sq/m^2^**  **vs. < 25** | **≥ 25** | **0.75** | **0.39－1.44** | **0.38** |  |  |  |
| **Smoking**  **vs. 0 cigarette per day** | **1－20**  **≥ 20** | **1.15**  **1.46** | **0.68－1.95**  **0.81－2.64** | **0.61**  **0.21** |  |  |  |
| **Alcohol consumption**  **vs. 0 mL of ethanol per day** | **0－25**  **≥ 25** | **0.75**  **0.85** | **0.36－1.56**  **0.47－1.55** | **0.44**  **0.60** |  |  |  |
| **Location of primary site**  **vs. Ut** | **Mt**  **Lt** | **1.62**  **1.41** | **0.64－4.07**  **0.53－3.79** | **0.31**  **0.49** |  |  |  |
| **LVLs**  **vs. A** | **B**  **C** | **2.10**  **2.57** | **1.30－3.39**  **1.15－5.72** | **0.003**  **0.02** | **2.12**  **2.74** | **1.31－3.42**  **1.22－6.18** | **0.002**  **0.02** |
| **Length of primary lesion**  **vs. <4 cm** | **≥ 4 cm** | **1.38** | **0.88－2.17** | **0.16** |  |  |  |
| **Treatment modality**  **vs. Surgery** | **CRT** | **1.44** | **0.92－2.24** | **0.11** | **1.51** | **0.96－2.37** | **0.08** |

^a^ The patients included in these analyses had no missing data regarding the baseline background

*HR* hazard ratio, *CI* confidence interval, *ECOG PS* Eastern Cooperative Oncology Group performance status, *Ut* upper thoracic esophagus, *Mt* middle thoracic esophagus, *Lt* lower thoracic esophagus, *LVLs* Lugol-voiding lesions, *CRT* chemoradiotherapy

**Supplementary Table 3. Univariate and multivariable analysis of the development of head and neck cancers** **(n= 284 patients)^a^**

| **Factors** |  | **Univariate analysis** | | | **Multivariate analysis** | | |
| --- | --- | --- | --- | --- | --- | --- | --- |
|  |  | **HR** | **95% CI** | ***P*** | **HR** | **95% CI** | ***p*** |
| **Age, years**  **vs. < 65** | **≥ 65** | **0.46** | **0.20–1.04** | **0.06** | **0.25** | **0.10–0.63** | **0.004** |
| **Gender**  **vs. female** | **Male** | **2.37** | **0.56－10.04** | **0.16** | **2.72** | **0.79－9.35** | **0.11** |
| **ECOG PS**  **vs. 0** | **1** | **3.47** | **0.40－29.95** | **0.26** | **2.51** | **0.29－21.93** | **0.41** |
| **Body mass index**  **vs. < 25, sq/m^2^** | **≥ 25** | **0.85** | **0.29－2.50** | **0.77** | **1.25** | **0.39－4.00** | **0.70** |
| **Smoking**  **vs. 0 cigarette per day** | **1－20**  **≥ 20** | **0.73**  **1.36** | **0.29－1.81**  **0.54－3.44** | **0.50**  **0.52** | **0.63**  **1.47** | **0.18－2.20**  **0.41－5.24** | **0.46**  **0.55** |
| **Alcohol consumption**  **vs. 0 mL of ethanol per day** | **0－25**  **≥ 25** | **1.05**  **0.83** | **0.34－3.29**  **0.31－2.19** | **0.93**  **0.70** | **1.39**  **0.37** | **0.35－5.58**  **0.11－1.22** | **0.65**  **0.10** |
| **Location of primary site**  **vs. Ut** | **Mt**  **Lt** | **1.35**  **1.08** | **0.31－5.86**  **0.22－5.41** | **0.69**  **0.92** | **1.55**  **0.92** | **0.31－7.81**  **0.18－4.61** | **0.59**  **0.92** |
| **LVLs**  **vs. A** | **B**  **C** | **7.62**  **10.92** | **2.26－25.69**  **2.56－46.62** | **0.001**  **0.001** | **8.92**  **18.97** | **2.49－32.02**  **3.86－93.17** | **<0.001**  **<0.001** |
| **Length of primary lesion**  **vs. <4 cm** | **≥ 4cm** | **1.85** | **0.87－3.94** | **0.11** | **1.67** | **0.75－3.69** | **0.21** |
| **Treatment modality**  **vs. Surgery** | **CRT** | **1.52** | **0.72－3.22** | **0.27** | **2.65** | **1.05－6.71** | **0.04** |

^a^ The patients included in these analyses had no missing data regarding the baseline background

*HR* hazard ratio, *CI* confidence interval, *ECOG PS* Eastern Cooperative Oncology Group performance status, *Ut* upper thoracic esophagus, *Mt* middle thoracic esophagus, *Lt* lower thoracic esophagus, *LVLs* Lugol-voiding lesions, *CRT* chemoradiotherap

**Supplementary Table 4. the relationship between LVL grades and SPMs of each organ** **(n= 379 patients)**

| **Factors** | **LVLs** | **Event N / Total N** | **%** | **Univariate analysis** | | |
| --- | --- | --- | --- | --- | --- | --- |
|  |  |  |  | **HR** | **95% CI** | ***p*** |
| **Head and Neck** | **A**  **B**  **C**  **Unknown** | **4 /170**  **21 /136**  **6 /30**  **4 /43** | **2%**  **15%**  **20%**  **9%** | **1**  **6.49**  **8.78**  **3.87** | **2.34－18.84**  **2.42－31.81**  **0.97－15.51** | **<0.001**  **<0.001**  **0.06** |
| **Stomach** | **A**  **B**  **C**  **Unknown** | **11/170**  **10/136**  **0/30**  **3/43** | **6%**  **7%**  **0%**  **7%** | **1**  **1.10**  **NE**  **1.0** | **0.47－2.60**  **0.29－3.52** | **0.83**  **0.99** |
| **Lung** | **A**  **B**  **C**  **Unknown** | **9/170**  **5/136**  **1/30**  **1/43** | **5%**  **4%**  **3%**  **2%** | **1**  **0.67**  **0.55**  **0.39** | **0.23－2.01**  **0.07－4.54**  **0.05－3.08** | **0.48**  **0.58**  **0.37** |
| **Urinary tract** | **A**  **B**  **C**  **Unknown** | **2/170**  **6/136**  **0/30**  **1/43** | **1%**  **4%**  **0%**  **2%** | **1**  **3.67**  **NE**  **1.83** | **0.75－18.01**  **0.16－20.49** | **0.11**  **0.62** |
| **Colorectal** | **A**  **B**  **C**  **Unknown** | **3/170**  **5/136**  **0/30**  **1/43** | **2%**  **4%**  **0%**  **2%** | **1**  **2.06**  **NE**  **1.22** | **0.49－8.70**  **0.12－12.23** | **0.33**  **0.87** |

*HR* hazard ratio, *CI* confidence interval, *LVLs* Lugol-voiding lesions, *NE* not evaluated
