# Supplementary material for: Second primary malignancies in patients with clinical T1bN0 esophageal squamous cell carcinoma after definitive therapies: supplementary analysis of the JCOG trial: JCOG0502
Source: J Gastroenterol. 2022 May 11;57(7):455–63. doi: 10.1007/s00535-022-01870-y (PMC9232445; doi:10.1007/s00535-022-01870-y)
Supplement: Supplementary file 2 — Supplementary file2 (PPTX 69 KB) [file 535_2022_1870_MOESM2_ESM.pptx]

## Slide 1
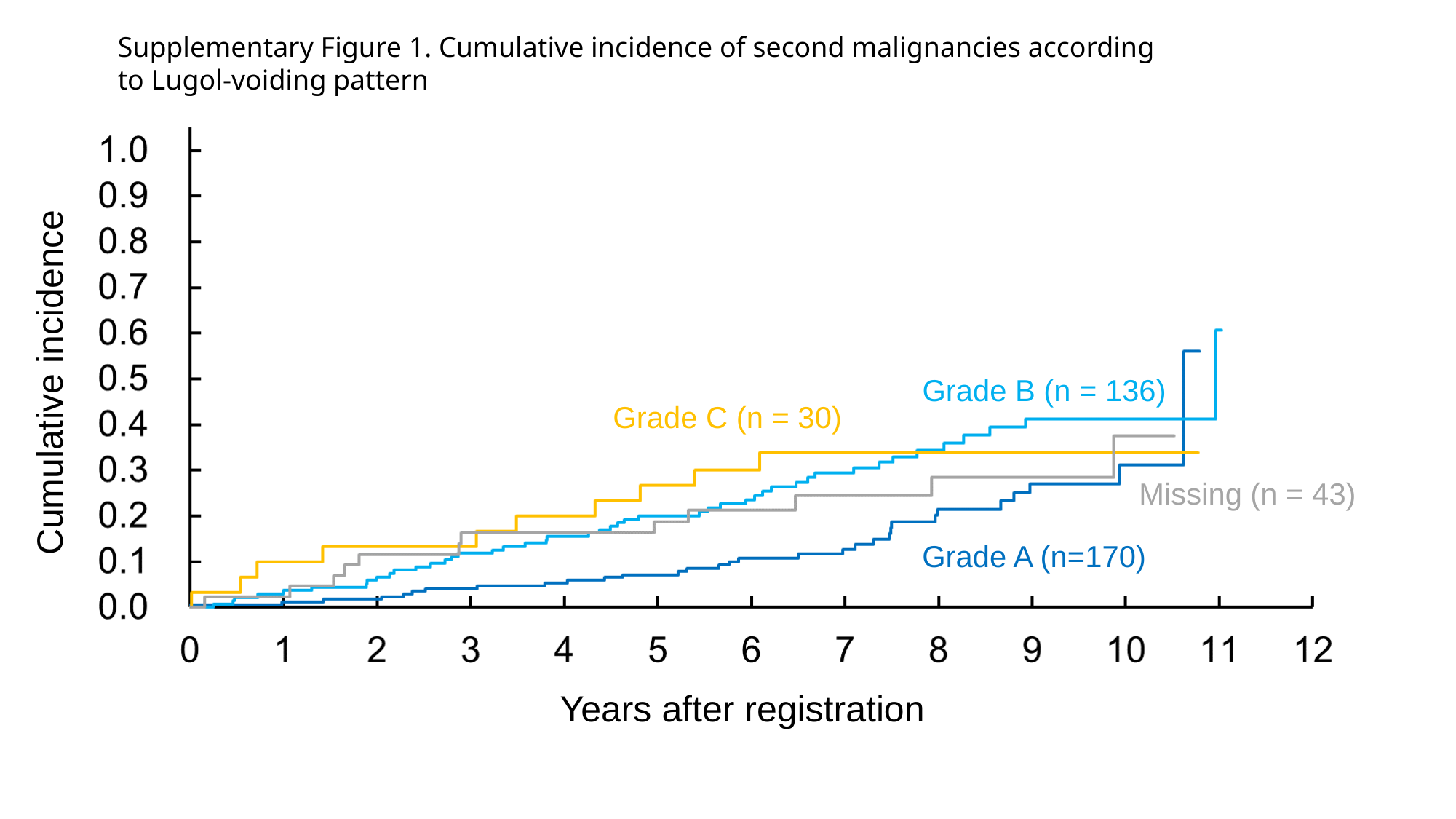

Supplementary Figure 1. Cumulative incidence of second malignancies according to Lugol-voiding pattern
Cumulative incidence
Grade B (n = 136)
Grade C (n = 30)
Missing (n = 43)
Grade A (n=170)
Years after registration
